# The Impact of Type of Brandy on the Volatile Aroma Compounds and Sensory Properties of Grape Brandy in Montenegro

**DOI:** 10.3390/molecules27092974

**Published:** 2022-05-06

**Authors:** Danijela Raičević, Tatjana Popović, Dejan Jančić, Danijela Šuković, Radmila Pajović-Šćepanović

**Affiliations:** 1 Biotechnical Faculty, University of Montenegro, Mihaila Lalica 1, 81000 Podgorica, Montenegro; tatjana.popovic@ucg.ac.me (T.P.); radmila.pajovic@ucg.ac.me (R.P.-Š.); 2 LLC Center for Ecotoxicological Research Podgorica, Bulevar Šarla de Gola 2, 81000 Podgorica, Montenegro; dejan.jancic@ceti.co.me (D.J.); danijela.sukovic@ceti.co.me (D.Š.)

**Keywords:** grape brandy, Muscat brandy, Kratošija, Muscat grape varieties, Vranac, volatile aroma compounds, sensory properties

## Abstract

This paper presents the results of a study that examined the impact of grape variety on the volatile aroma compounds and sensory properties of standard and Muscat grape brandy produced in the Podgorica sub-region (Montenegro) in vintages 2011, 2012, and 2013. The brandies were prepared by the distillation of crushed grapes, from the autochthonous varieties of Vranac and Kratošija, and Muscat grapes, in a traditional copper alembic, under the same conditions. The gas chromatographic-mass spectrometric (GC/MS) method of 82 volatile aroma compounds that belong to the group (alcohols, volatile acids, volatile esters, terpenes, volatile aldehydes, acetals, ethers, ketones, and alkanes) and an evaluation of the sensory properties of brandies were carried out to determine the typical characteristics of the examined brandies. Alcohols, fatty acid esters, and terpene compound contents were significantly more abundant in all Muscat grape brandies compared to the brandies from the Vranac and Kratošija wine varieties (Standard brandy). Research results revealed that variety had a significant impact on the volatile aroma compound and sensory properties of brandy. The varietal effect was also confirmed, by multivariate analysis, based on the aroma volatile composition, which showed a grouping by type of grape brandy (varietal origin). Sensory analyses showed that all the brandies belonged to the category of high-quality brandies.

## 1. Introduction

Grape brandy or brandy is obtained through the fermentation and distillation of grape pomace around the noble grape vine *Vitis vinifera* [1,2,3]. The quality of grape brandies depends on many factors: the variety of grape, climate, land and agricultural practices, yield, fermentation characteristics, timing and distillation methods, storage methods, and other distillates [4,5,6,7]. According to Montenegro’s law regarding spirit drinks (2016) [8], fruit brandies, the category to which grape brandy belongs, must contain at least 37.5% vol. of alcohol (ethanol).

Aromatic volatile compounds (alcohols, volatile acids, volatile esters, terpenes, volatile aldehydes, and acetals) are important sensory indicators and quality parameters for alcoholic beverages [9]. The varietal aroma potential of grapes is one of the most important factors determining the brandy’s character, quality, and typicity, particularly in Muscat-based brandies [10].

Higher alcohol contents are interesting in terms of their great influence on the quality of brandy, as they contribute to its body and aromatic complex, so they only are undesirable if present in excessive concentrations [11,12]. In brandies, the most abundant alcohols are associated with oily, green, cut grass; grass and herbaceous aromas (1-hexanol); and rose and honey aromas (phenyl-ethyl alcohol), while 1-heptanol is associated with an oily aroma [13,14,15,16,17].

Terpenes are the main group of aromatic substances for grapes (*V. vinifera*), wine, and distillates of Muscat varieties, and among them, the most important are linalool, geraniol, citronellol, and nerol, but also terpineol. Terpenes are mainly responsible for fine aromatic and floral aromas and represent the most important factor affecting the aroma in Muscat and non-Muscat cultivars [17]. Esters are compounds with ingredients that most frequently contribute to pleasant, fruity, and floral aromas in brandy [13,17]. Medium-chain fatty acid ethyl esters (hexanoic, octanoic, decanoic, and dodecanoic) are particularly important because they have a high positive effect on aroma, low odour thresholds, and provide fruity and floral notes [11,12,13,17,18,19]. Fatty acids are known to play an important role in the sensory quality of beverages. They contribute to flavour, as a precursor of volatile compounds. Fatty acids are associated with a number of aroma groups, including esters [11,12,17].

Aldehydes, which represent a rather widely described class of compounds, as aroma compounds, are often noted to have a negative influence on the quality characteristics of spirits [19]. Studies show that total aldehyde concentrations higher than 1200 mg/L (mainly acetaldehyde) significantly affect the aroma of the distillate and indicate a significant oxidation of ethanol during fermentation [16,19,20].

Acetals contribute to the aroma of many alcoholic beverages that are derived from fruit. They give alcoholic beverages a delicate pleasant taste and bouquet [20,21,22].

The sensory properties of alcoholic beverages are influenced by a high number of compounds, which are present in small amounts but have a large influence on the bouquet. The most important are alcohols, terpenes, and esters.

In Montenegro, there are favourable environmental conditions and a long tradition of growing wine and table grapes and producing wine and brandy [23,24,25]. The Vranac variety dominates wine and brandy production (80%), followed by Kratošija and other international varieties [26,27,28]. Wine and table grape varieties show significant differences in their morphological and production characteristics. Regarding technological characteristics, the table grape varieties are usually aromatic or Muscat, which provides added value because the aroma, when converted to distillate, is the most appreciated feature [29,30]. Grape brandy is a traditional Montenegrin product, mainly produced in southern and central Montenegro. Among grape brandies, standard and Muscat grape brandy of the Biotechnical Faculty, known as the Institute’s grape brandy, have an excellent reputation, with a long manufacturing tradition. To our knowledge, there is no study on grape brandy quality in Montenegro.

In the present study, we examined the quality of the Montenegrin standard grape brandy and Muscat grape brandy produced by the Biotechnical Faculty over 3 years of harvest to determine their quality standards and the influence of grape variety on the quality of the brandy.

## 2. Materials and Methods

### 2.1. Chemicals

Ultra-high-purified water and methylene chloride HPLC grade, methyl 10-undecenoate, anhydrous sodium sulfate, and sodium chloride were purchased from Sigma-Aldrich (Steinheim, Germany). Ultra-high-purified methylene chloride HPLC grade from Merck (Darmstadt, Germany) was used as the solvent for extraction.

### 2.2. Grape Marc Samples

Three grape varieties (Vranac, Kratošija, and Muscat grapes) were harvested from the experimental vineyard, owned by the Biotechnical Faculty, located in the Lješkopolje district or the Podgorica sub-region. The grapes were sampled at the optimal technological maturity (22.5–23.7 Brix) for wine grape varieties and 21.8–22.2 Brix for Muscat table wine varieties, between 1 and 29 September in 2011, 2012, and 2013.

Standard grape brandy is produced from the autochthonous wine grape varieties Vranac (70%) and Kratošija (30%). Muscat grape brandy is made from table Muscat varieties (Muscat Hamburg, Muscat Italy, and Afus Ali), which are dominant in the production of this brandy and Muscat grape wine varieties (collection of 400 different genotypes), but in negligible quantities.

The grapes were mashed by a stalk-removing electric crusher. The alcoholic fermentation of the pomace was performed in 500 kg containers in the conventional way, with spontaneous fermentation triggered by the indigenous microflora of wine yeasts.

### 2.3. Distillation of Fermented Grape Marc Samples

The distillation process was performed using a discontinuous Sharant-type copper apparatus of 250 L volume (Mandarić, Novi Sad, Serbia) Uniform and regular distillations were achieved by gentle heating of the alembic at the beginning of distillation, which became stronger during the main stream in the distillation and quieting at the end of distillation. The water temperature in the cooling tank was kept between 20 °C and 22 °C. Depending on the raw materials, the distillation lasted between 2 and 3 h. During the distillation of fermented material, three basic fractions were singled out: the first (the head), middle-run (the heart fraction), and the tail fraction. The middle-run part was saved as a fresh distillate. Distillates were stored in dark stoppered bottles at 20 °C for 12 months.

### 2.4. Analysis of the Chemical Quality Parameters of Grape Brandy

According to the official Montenegrin legislation on methods for sampling and performing chemical and physical analyses of alcoholic beverages [31], the following analyses were performed:

The alcoholic strength by volume was determined after distillation using a pycnometer [32].

The methanol content was analysed on the basis of the European Community Reference Methods for the analysis of spirits using gas chromatography (GC) with a flame-ionization detector (FID). Methanol was determined by the direct injection of a sample into a gas chromatography (GC) system (GC 2010 plus, Shimadzu, Japan). As an internal standard, 2-butanol was added to the spirit drink prior to injection. Methanol was separated by temperature programming on SPB^®^-624 Capillary GC Column (30 m × 0.25 mm, df 1.40 μm) and detected using a flame ionisation detector (FID). The methanol concentration was determined with reference to the internal standard from response factors, which were obtained during calibration under the same chromatographic conditions as those of the spirit drink analysis. [32].

#### 2.4.1. Extraction and Concentration of Minor Volatile Constituents

Fifty millilitres of distillate were mixed with 100 mL of ultrapure water, 20 mL of internal standard concentration 1 mg/L (methyl 10-undecenoate) was added, and then it was extracted with 40 mL of dichloromethane. NaCl (10 g) was added, and the mixture was stirred magnetically for 30 min. Layers were separated in a separator funnel, and the organic layer was dried (2 h) over anhydrous sodium sulphate. The extract was concentrated to 1.0 mL under nitrogen and directly analysed on GC/MS.

#### 2.4.2. GC/MS Analysis of Minor Volatile Compounds

GC/MS analysis was performed using an Agilent 6890 gas chromatograph (Agilent Technologies, Santa Clara, CA 95051, USA) coupled with Agilent 5973 Network mass selective detector (MSD) operated in the positive ion electron impact (EI) mode. The separation was achieved on an Agilent 19091S-433 HP-5MS-fused silica capillary column, 30 m × 0.25 mm i.d., 0.25 μm film thickness. The GC oven temperature was programmed from 60 to 300 °C at a rate of 3 °C/min. Helium was used as the carrier gas, the inlet pressure was 25 kPa, and the velocity was 1 mL/min at 210 °C. The injector temperature was 250 °C and the injection mode was split less. The MS scan conditions were source temperature, 200 °C; interface temperature, 250 °C; energy of the electron beam was 70 eV; and the mass scan range was 40–350 amu (atomic mass units). The identification of the components was based on retention indices and comparison with reference spectra (Wiley and NIST databases). For quantitative evaluation, methyl 10-undecenoate was used as an internal standard (IS).

### 2.5. Sensory Analysis

The sensory evaluation of the spirit samples was performed using a Buxbaum model with a positive rating of 20, which is the worldwide-accepted method for the sensory evaluation of strong alcoholic drinks. This model is based on five sensorial experiences: colour, clarity, distinction, odour, and taste. This is the most common method used in Western Balkan countries [33,34,35,36].

The tasting panel was composed of a group of 15 tasters, including 5 licensed and 10 unlicensed evaluators. The licensed tasters completed the training and passed the aptitude test of tasters for organoleptic assessment of spirits at the Biotechnical Faculty in Ljubljana, Slovenia.

The tasters were asked to score sensory attributes with a structured scale: colour and clarity (maximum 1), distinction (maximum 2), odour (max 6), and taste (max 10). The evaluation was performed in the tasting room, Biotechnical Faculty, University of Montenegro. For successful evaluation, all conditions were provided, including: space, air temperature 22 ± 1 °C, white light source, tasting utensils, etc. To achieve uniform taste and odour, the samples, 30 mL, were tasted at 12/16 °C, in colourless, transparent glass with a tulip-shaped (ISO 3591:1977). The samples were coded and tested in the balanced order to eliminate the first order carry-over effect and evaluated on the same day. The standard grape brandy was evaluated before Muskat grape brandy, because of an expected stronger aroma in Muskat ones. Water was provided for mouth-rinsing between samples.

### 2.6. Statistical Analysis

Analysis of the experimental data was performed using the statistical package IBM SPSS Statistics 20 (IBM Corporation, New York, NY, U.S.A.). Analyses of the differences between the two types of grape brandies from three vintages, as well as their interactions, were carried out via a two-factor analysis of variance (ANOVA) and LSD test for an α = 5%.

The relationships between types of brandies were investigated by principal component analysis (PCA). The PCA was based on the aroma composition of the examined brandies. Tree replicates were used to create a correlation matrix from which standardized principal component (PC) scores were extracted. Scatter plots of the first three PCs were created with Statistic/Graph. To determine which of the PCs accounted for the greatest amount of variation, the eigenvalues of the two PCs were compared for each trait. Data processing was performed using the statistical programme Statistica, version 8 (StatSoft, Inc., Tulsa, OK, U.S.A.).

## 3. Results and Discussion

### 3.1. Volatile Compounds of Brandy Samples

Using the combined gas chromatographic–mass spectrometric method (GC/MS), in the examined brandy samples, 82 volatile aromatic compounds were found, which belonged to different groups, such as a higher alcohols, esters, acids, terpenes, aldehydes, acetals, and other compounds. For all brandies, esters were the most-represented chemical class, followed by alcohols [37].

#### 3.1.1. Alcohols

The average alcoholic strength by volume (ethanol) of standard grape brandy reached the value of 50.1% vol., while the alcoholic strength of Muscat grape brandy was 49.8% vol. There was no statistically significant difference in alcohol content between the tested brandies.

The methanol content in the analysed samples ranged from 125 g/hL of 100% vol. alcohol in the standard grape brandy to 175 g/hL of 100% vol. alcohol in Muscat grape brandies. The levels for our samples were found to be much lower than those given in the European legislation (Regulation (EU) 2019/787) and law on spirit drinks in Montenegro (2016) [8], where a methanol concentration lower than 1000 g/hL of 100% vol. alcohol was indicated. No statistical significance was confirmed for the differences. These methanol values indicate the proper manipulation of the raw material with well-performed distillation procedures [13,21,36]. The methanol contents in analysed samples are in the same range (95.5–259 g/L) as the results presented for grape brandy [15,30,33,38], but lower than the content given for other fruit brandies [13,21,36,39,40].

In our research, higher alcohols were quantitatively the most abundant group of volatile compounds, which confirms the research findings [11,22], stating that these components give the specific aroma, taste, and basic character of alcoholic beverages.

Among all the examined higher alcohols (presented in the Table 1) in examined brandies, 2-phenylethyl alcohol and 1-hexanol had the highest contents.

A higher average content of phenyl-ethyl alcohol was detected in Muscat brandy samples (6.76 mg/ L), in comparison with standard brandies (5.98 mg/L). The highest content was recorded in the sample M1 (7.14 mg/L), and the lowest in the sample S3 (5.78 mg/L). The results are in line with [13,15,17,19].

The average 1-hexanol content in Muscat brandies is 1.94 mg/L, while it is 1.12 mg/L in standard grape brandies. A significantly higher relative content of 1-hexanol in relation to other brandies was found in distillate M1 (3.30 mg/L). The results are in line with the findings of Apostolopoulou et al. and Cacho et al. [14,41]. When compared to other fruit brandies, 1-hexanol was detected in lower concentrations in the examined samples [15,17,19]. This can be explained by the use of well-ripened grapes and weaker pressing during the preparation of grapes for fermentation, which probably affected the good quality of brandy. A higher average content of 1-heptanol was found in Muscat brandies (0.37 mg/L) in relation to the standard ones (0.14 mg/L).

#### 3.1.2. Esters

All examined ester compounds are presented in Table 2.

The results of the total volatile esters identified in this study show that their average content in Muscat brandy (25.18 mg/L) is statistically significantly higher than the content in standard brandies (20.49 mg/L). The most abundant esters in all samples are medium chain-fatty acid ethyl esters. Among these, the most common are ethyl decanoate (5.81 mg/L), in line with the previous research by Christoph et al., Matijašević et al., Hernández-Gómez et al., and Matias-Guiua et al. [11,17,18,42]; followed by ethyl octanoate (3.40 mg/L), ethyl dodecanoate (2.86 mg/L), and ethyl hexanoate (2.54). Ethyl decanoate, ethyl hexanoate, and ethyl octanoate provide a pleasant fresh tropical fruit aroma, while ethyl dodecanoate gives a pear aroma and a characteristic fruit aroma. [17,42].

The ethyl decanoate was higher in Muscat brandies, with an average content of 6.18 mg/L (maximum concentration of 7.14 mg/L in sample M1), compared to the standard ones (5.43 mg/L). The average content of ethyl octanoate was three times higher in Muscat brandies (5.16 mg/L) than in standard brandies (1.64 mg/L). The average content of ethyl dodecanoate was 2.94 mg/L in Muscat brandies and 2.78 mg/L in standard brandies. The average content of 2.94 mg/L of ethyl hexanoate was recorded in standard brandies, and 2.14 mg/L was recorded for Muscat brandies. A strong correlation was observed between the total intermediate chain acids and the corresponding ethyl esters, so it was assumed that their amounts were significantly predetermined by the acid precursor content [19].

Much lower diethyl succinate concentrations (average content was 2.36 mg/L) were present in our samples than in other studies of Soufleros et al. [13] (3.19 mg/L), Cortés et al. [22] (23.5 mg/L), and Schreier et al. [43] (German brandy 10.2 mg/L, French brandy 6.4 mg/L).

The ethyl esters of long-chain fatty acids usually do not have a significant effect on the aroma of alcoholic beverages, unless they are present in extremely large quantities [19,21]. We identified the highest average content of ethyl hexadecanoate (1.18 mg/L) and ethyl 9,12-octadecadienoate (1.03 mg/L).

#### 3.1.3. Terpenes

A list of the analysed terpenes in the examined brandies is given in Table 3. The results of the investigation showed that the average content of terpenes was three times higher in Muscat brandies (1.98 mg/L) in comparison with standard brandies (0.65 mg/L), while some research reported it to be up to four times higher [44].

Linalool is the major component of all the examined samples, in accordance with other research findings [41]. This is followed by *E*-nerolidol, (*2Z*,*6E*)-farnesol, citronellol, geraniol, and alpha-terpineol. The average content of linalool, which significantly contributes to the aroma of roses, anise seeds, grapefruit, lime, and citrus, is as much as 10 times higher in Muscat brandy (0.50 mg/L) compared to standard brandy (0.05 mg/L). This means that the average concentration of linalool in Muscat brandies is significantly above the threshold of perception (0.05 mg/L) [45]. The obtained values are in accordance with the research [17,43,45,46].

Terpene *E*-nerodilol mainly represents floral and citrus flavours [9], and its average content in the examined samples is 0.24 mg/L, which is under the threshold of perception (0.4 mg/L) [45]. The average content of farnesol in tested brandy is 0.20 mg/L.

The average content of citronellol, which is, together with linalool, one of the most important terpenic compounds, is 0.10 mg/L. This value is in line with [45] (0.07–43.53 mg/L) and significantly above the threshold of perception (0.018 mg/L).

The content of citronellol is almost three times higher in Muscat brandies (0.14 mg/L) than in standard brandies (0.05 mg/L). The average geraniol content of 0.17 mg/L in Muscat brandies and 0.07 mg/L in standard brandies will also contribute to floral, and especially rose aromas, because its concentration is above the threshold of perception (0.13 mg/L), as determined by [45]. Alpha-terpineol provides the aromas of flowers, iris, and pine forest; 0.14 mg/L was detected in Muscat brandy, and 0.05 mg/L in standard brandy.

Our results confirmed the conclusions of [12,17,45,47], that the aroma of Muscat grape (brandy) is directly related to different monoterpenes, such as linalool, rose oxide, citral, nerol, geraniol, and citronellol.

#### 3.1.4. Volatile Acids

The average content of total fatty acids, shown in Table 4, is approximately the same in Muscat (5.28 mg/L) and standard brandies (5.05 mg/L). For medium-chain fatty acids, decanoic, octane, dodecane, and hexane were identified in all brandy samples. Medium-chain fatty acids usually do not show a significant effect on the aroma of the distillate due to the relatively high odour perception thresholds of 8 and 15 mg/L determined for decanoic and octanoic acid, respectively [19]. Table 4 shows that decanoic acid has the highest average value (2.01 mg/L) for all the tested samples of distillates (standard and Muscat brandies) compared to other fatty acids. This is followed by octanoic acid (0.84 mg/L), dodecanoic acid (0.66 mg/L), and hexanoic acid (0.61 mg/L), which is in accordance with the research [11,17,19,38].

Regarding the long-chain fatty acids, which, with glycerol, contribute to the aroma and the body (oiliness and viscosity) of the beverage [48], hexadecanoic acid (palmitic) has the highest average value in all tested samples (0.43 mg/L), which is in line with [17,19,38].

#### 3.1.5. Carbonyl Compound (Aldehydes and Acetals)

The concentrations of aldehydes, acetals, and other compounds in standard and Muscat grape brandies are given in Table 5.

As expected, the major carbonyl compound in distillates is acetaldehyde, a direct alcoholic fermentation by-product, which usually accounts for 90% of the total aldehyde contents [16,21].

There was no statistically significant difference between the average acetaldehyde content in standard grape brandy (209 mg/L of 100% vol. alcohol) and in Muscat brandy (215 mg/L of 100% vol. alcohol). These results are in accordance with the results described in the literature [18,19,38]. The values we found in this study are lower compared to the ones reported earlier [15,22,39], and significantly lower than the official limits for fruit distillates adopted by the European Council (Regulation (EU) 2019/787) [49].

A higher concentration of benzaldehyde was found in standard brandies (0.21 mg/L) than in Muscat brandies (0.17 mg/L). The benzaldehyde content, which contributes to the taste of bitter almonds, marzipan, and cherries, identified in the tested brandy, is in accordance with the literature data [11,43,46,47,48]. However, it is significantly lower than the values obtained earlier [15,19,22,39,47]. Benzaldehyde is not desirable in large amounts, but, when present in small amounts, it contributes to the complexity of flavour.

The content of furfural in the analysed samples ranges from 1.4 to 5.0 mg/L of 100% vol. alcohol and is significantly lower than the perception threshold of this compound, which is 150 mg/L. Approximately the same average content was found in both types of brandy. Furfural is a normal ingredient in fruit and can be used as an indicator of distillate naturalness, but the increased concentration of it may contribute to the presence of “roasted” tones in brandy [12,16]. These results are in line with the available literature results [12,15,18,19,33,39,41]. The average content of nonanal in the tested brandies is 0.045 mg/L.

We also investigated acetals compounds 1,1-diethoxy-2-methylpropane, 3,3-diethoxy-2-butanone, and 1-(1-ethoxyethoxy) pentane (Table 5). There were no statistically significant differences in the acetal compounds’ average value in standard (0.36 mg/L) and Muscat brandies (0.34 mg/L). The acetal content and rather low acetaldehyde content, in all the samples, indicate the correct technological process, with regular fermentation without oxidative events [16,20,21].

### 3.2. Sensory Analysis

The sensory characteristics of the examined brandies are presented in Table 6.

The produced brandies are colourless and clear, and distinguished by the odour and taste characteristic of grape brandies. The examined brandies scored a maximum number of points (1.0) for colour and clarity. The Muscat brandy obtained a higher average score for odour (5.74), as Muscat grape varieties provide a distinctive Muscat aroma and strong fruity taste. The Standard grape brandy had a higher average score for taste (9.48) because of its drinkability, full flavour, and harmony. The average sensory score between the standard (19.0) and Muscat grape brandies (18.8) differed by only 0.2 points, meaning that, in terms of sensory evaluation, the two brandies were of similar quality, as confirmed by the ANOVA test. The results for the total score of the investigated grape brandies were much higher than grape brandies produced in Serbia, where authors used the same score method (BuxBaum) for sensory evaluation [33,34,35,36]. The average values were between 17.11 and 17.48 [33] and between 15.07 and 174 [35]. The presented results for Montenegrin grape brandies were also higher in comparison to the fruit brandy: 17.60 to 18.40 [36] and 18.1 to 18.6 [34]. These results indicate that their extraordinary odour, exceptional drinkability, and harmony make both types of brandies unique Montenegrin brands.

### 3.3. Classification of the Type of Grape Brandy Based on Aroma Composition

Principal component analysis (PCA) was applied to obtain further information on what affects the different contents of aroma compounds of the examined grape brandies: whether it is the different type of brandies (varietal origin of grape) or vintage year. A correlation matrix was created based on the aromatic composition of grape brandies in terms of higher alcohols (1-hexanol, 1-heptanol, 2-phenylethyl alcohol), esters (ethyl hexanoate, ethyl succinate, ethyl octanoate, ethyl decanoate and ethyl dodecanoate), terpenes (linalool, alpha-terpineol, citronellol, geraniol, *2Z*,*6E*-farnesol and *E*-nerolidol), acids (hexanoic acid, octanoic acid, decanoic acid, dodecanoic acid and hexadecanoic acid), and aldehydes (benzaldehyde and nonanal).

The PCA results indicated that the first principal component (PC1) accounts for 64.24%, the second principal component (PC2) accounts for 11.69%, the third principal component (PC3) accounts for 8.58%, and the fourth principal component (PC4) accounts for 4.97% of the variability. These four components were extracted, since they had eigenvalues greater than 1.0. Together, they account for 89.49% of the variability in the original data.

**Figure 1:**
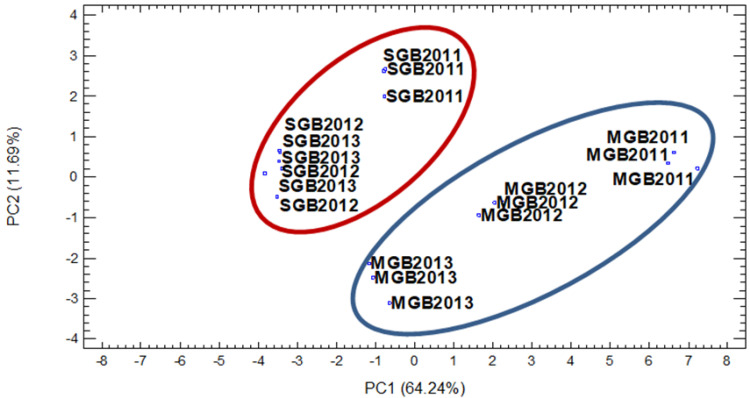
The scatter plot of distribution compounds of studied brandies: Muscat grape brandy (MGB) and standard grape brandy (SGB) for three consecutive vintages.

**Figure 2:**
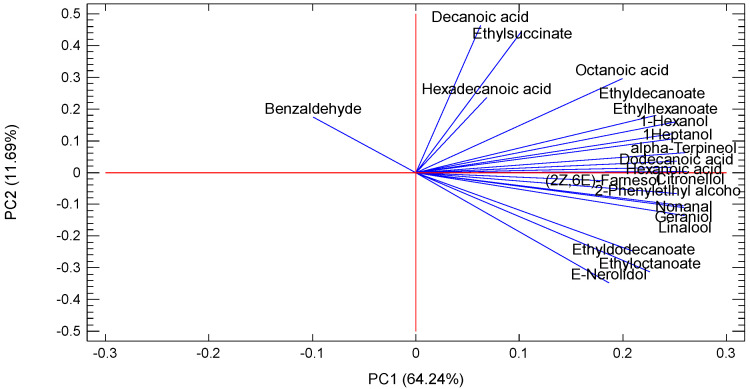
Factor score for four principle components of brandies.

The correlation between the original variables and the first four principal components is shown in Figure 1. Based on the position of samples on the scatter plot, it can be observed that grape brandies were separated into two distinct groups according to type of brandies and grape variety. Thus, the samples of standard grape brandies (SGB) were located in the negative region of PC1. Samples of Muscat grape brandies (MGB) were located in the positive region of PC1 except for samples from vintage 2013, which were located in the negative part for PC2. The separation of the two types of grape brandies was based on the higher content of some of the groups’ aromatic compounds: there were more alcohols, esters, and terpenes in Muscat grape brandies. All these compounds were located in the positive part of PC1 on the plot of component weight (Figure 2). The content of ethyl succinate decanoic acid and benzaldehyde caused separation of SGB due to the increased content of these compounds in comparison to MGB. This clear separation of different types of brandies was based on the different contents of the aforementioned compounds and aroma groups, and confirmation data are presented in Table 1, Table 2 and Table 3 and the following comments.

Regarding the examined vintage year, we could not find a grouping for grape brandies based on this criterion. We can note an exception for Muscat brandies with the vintage 2011 (MGB 2011), which were located in the positive region of PC1 and PC2 due to their having the highest content of some compounds in the groups of higher alcohols, esters, terpenes, and acids.

The groupings presented in the PCA plots confirm the conclusion of the previous section regarding the ability to group the grape brandies according to the grape variety used for brandy production.

## 4. Conclusions

Given the same production technology, the average ethanol, methanol, volatile acids, aldehydes, and acetals contents were approximately the same in the examined brandies. The volatile aroma compounds content (higher alcohols, volatile esters, and terpenes) was higher in Muscat than in standard grape brandy, considering the higher percentage of the aromatic compounds in the Muscat grapes used for the production of this type of brandy.

The results of our research showed that the grape variety used for brandy production influences some sensory parameters (odour and taste), but there were no statistically significant differences between the average total marks for different types of brandies. Sensory analyses showed that all brandies belonged to the category of high-quality brandies and were of uniform quality.

Principle component analyses showed good groupings by type of brandy according to aroma composition. They confirmed a clear separation between standard grape brandies and Muscat grape brandies.

The research findings indicate the high quality (volatile aroma compounds and sensory properties) of the investigated brandies, making them a truly distinctive brand in Montenegro.

## Figures and Tables

**Table 1:** Concentration of alcohols in standard and Muscat grape brandies in mg/L *.

Compound	Standard Grape Brandy (SGB)			Muscat Grape Brandy (MGB)		
	S1	S2	S3	M1	M2	M3
Ethanol (%vol.)	49.9 ± 1.4	50.1 ± 1.0	50.2 ± 1.0	49.8 ± 0.6	49.7 ± 0.8	49.9 ± 0.9
Methanol (g/hL of 100% vol. alcohol)	149 ± 10	125 ± 5	170 ± 6	161 ± 8	129 ± 8	175 ± 10
Aroma compound (mg/L)						
1-hexanol **	1.18 ± 0.06 ^c^	1.06 ± 0.08 ^c^	1.10 ± 0.15 ^c^	3.30 ± 0.17 ^a^	1.82 ± 0.05 ^b^	0.71 ± 0.05 ^d^
4-heptenol-1ol	0.03 ± 0.01	0.010 ± 0.002	0.020 ± 0.004	0.05 ± 0.01	0.020 ± 0.003	0.020 ± 0.002
1-heptanol **	0.21 ± 0.02 ^b^	0.11 ± 0.02 ^c^	0.10 ± 0.02 ^c^	0.75 ± 0.05 ^a^	0.25 ± 0.03 ^b^	0.11 ± 0.02 ^c^
1-octen-3-ol	0.11 ± 0.02	0.06 ± 0.01	0.050 ± 0.005	0.33 ± 0.01	0.16 ± 0.02	0.08 ± 0.01
*n*-octanol	0.17 ± 0.01	0.070 ± 0.003	0.060 ± 0.001	0.33 ± 0.01	0.19 ± 0.02	0.08 ± 0.01
*n*-decanol	0.12 ± 0.04	0.08 ± 0.01	0.09 ± 0.01	0.14 ± 0.02	0.06 ± 0.01	0.040 ± 0.003
1-tetradecanol	0.05 ± 0.02	0.050 ± 0.052	0.06 ± 0.08	0.07 ± 0.32	0.09 ± 0.07	0.030 ± 0.005
1-hexadecanol	0.040 ± 0.002	0.030 ± 0.005	0.030 ± 0.003	0.010 ± 0.001	0.04 ± 0.01	0.020 ± 0.002
3,7,11,15-tetramethyl-2-hexadecen-1-ol	0.040 ± 0.004	0.05 ± 0.01	0.06 ± 0.01	0.010 ± 0.002	0.010 ± 0.001	0.030 ± 0.003
1-methyl-cyclohexanol	0.15 ± 0.03	0.22 ± 0.03	0.14 ± 0.01	0.12 ± 0.01	0.07 ± 0.01	0.140 ± 0.001
3-ethyl-4-methylpentanol	n.d.	n.d.	n.d.	0.020 ± 0.002	n.d.	n.d.
benzyl alcohol	n.d.	0.160 ± 0.012	0.010 ± 0.002	n.d.	n.d.	n.d.
3,5,5-trimethyl-2-cyclohexen-1-ol	n.d.	n.d.	0.010 ± 0.001	0.010 ± 0.001	n.d.	n.d.
2-phenylethyl alcohol **	6.25 ± 0.08 ^c^	5.91 ± 0.04 ^d^	5.78 ± 0.07 ^e^	7.14 ± 0.09 ^a^	6.85 ± 0.05 ^b^	6.29 ± 0.08 ^c^
Total high alcohol	8.35 ± 0.17^c^	7.81 ± 0.14 ^d^	7.51 ± 0.13 ^d^	12.38 ± 0.31 ^a^	9.56 ± 0.10 ^b^	7.55 ± 0.11 ^d^
Average	7.89 ± 0.39 ^B^			9.80 ± 2.04 ^A^		

* data are presented as mean ± SD of three independent determinations; S1 (SGB vintage 2011), S2 (SGB vintage 2012), S3 (SGB vintage 2013), M1 (MGB vintage 2011), M2 (MGB vintage 2012), M3 (MGB vintage 2013); ** compounds in PCA analysis; n.d. = not detected; different superscript small letters in the same row indicate significantly different means (*p* < 0.05) for type of brandy from different vintages; different superscript capital letters indicate significantly different means (*p* < 0.05) for type of brandy.

**Table 2:** Concentration of esters in standard and Muscat grape brandies in mg/L *.

Aroma Compound (mg/L)	Standard Grape Brandy (SGB)			Muscat Grape Brandy (MGB)		
	S1	S2	S3	M1	M2	M3
Ethyl butyrate	0.190 ± 0.001 ^d^	0.140 ± 0.001 ^e^	0.14 ± 0.02 ^e^	0.23 ± 0.02 ^b^	0.16 ± 0.01 ^c^	0.18 ± 0.01 ^a^
Ethyl lactate **	0.40 ± 0.002	0.33 ± 0.03	0.34 ± 0.02	0.63 ± 0.02	0.52 ± 0.03	0.76 ± 0.01
Ethyl 3-methylbutyrate	0.020 ± 0.002	0.020 ± 0.002	0.020 ± 0.001	0.020 ± 0.003	0.020 ± 0.003	0.03 ± 0.01
Isoamyl acetate	0.160 ± 0.001	0.18 ± 0.02	0.12 ± 0.02	0.36 ± 0.03	0.30 ± 0.02	0.33 ± 0.02
Ethyl hexanoate **	2.960 ± 0.004 ^b^	1.86 ± 0.03 ^d^	1.60 ± 0.06 ^e^	3.69 ± 0.31 ^a^	2.95 ± 0.07 ^b^	2.19 ± 0.13 ^c^
Ethyl 2-hydroxy-4-methylpentanoate	0.030 ± 0.004	0.010 ± 0.002	0.020 ± 0.003	0.080 ± 0.004	0.040 ± 0.004	0.02 ± 0.01
Ethyl succinate **	2.57 ± 0.04 ^b^	2.49 ± 0.04 ^b^	2.25 ± 0.07 ^c^	2.80 ± 0.07 ^a^	2.04 ± 0.11 ^d^	2.00 ± 0.07 ^d^
Ethyl octanoate **	2.00 ± 0.09 ^d^	1.81 ± 0.04 ^e^	1.09 ± 0.05 ^f^	5.65 ± 0.08 ^a^	5.21 ± 0.04 ^b^	4.63 ± 0.06 ^c^
Ethyl nonanoate	0.06 ± 0.01	0.06 ± 0.01	0.05 ± 0.01	0.13 ± 0.02	0.03 ± 0.01	0.05 ± 0.01
Ethyl-(4*E*)-decenoate	0.14 ± 0.03	0.02 ±0.01	0.040 ± 0.003	0.040 ± 0.003	0.04 ± 0.01	0.020 ± 0.002
Ethyl decanoate **	6.48 ± 0.01 ^b^	4.87 ± 0.09 ^d^	4.95 ± 0.09 ^d^	7.14 ± 0.07 ^a^	5.75 ± 0.09 ^c^	5.64 ± 0.07 ^c^
Ethyl 3-methylbutyl succinate	0.03 ± 0.01	0.02 ± 0.01	0.020 ± 0.003	n.d.	0.030 ± 0.003	0.030 ± 0.003
Isoamyl octanoate	0.07 ± 0.01	0.030 ± 0.003	0.04 ± 0.01	0.050 ± 0.003	0.05 ± 0.01	0.020 ± 0.002
2-methylbutyl octanoate	0.020 ± 0.003	0.010 ± 0.002	0.010 ± 0.002	n.d.	0.020 ± 0.003	n.d.
Ethyl 9-oxononanoate	0.010 ± 0.002	n.d.	n.d.	0.070 ± 0.003	0.020 ± 0.002	0.140 ± 0.001
Isobutyl decanoate	0.010 ± 0.001	n.d.	0.010 ± 0.002	0.010 ± 0.001	0.010 ± 0.001	n.d.
Ethyl dodecanoate **	2.82 ± 0.04 ^c^	2.80 ± 0.03^c^	2.71 ± 0.04 ^d^	3.09 ± 0.08 ^a^	2.95 ± 0.05 ^b^	2.78 ± 0.04 ^cd^
Isoamyl decanoate	0.10 ± 0.01	0.06 ± 0.01	0.08 ± 0.03	0.050 ±0.001	0.050 ± 0.003	0.030 ± 0.003
2-methylbutyl decanoate	0.030 ± 0.003	0.020 ± 0.002	0.030 ± 0.003	0.020 ±0.003	0.020 ± 0.002	0.01 ± 0.01
Ethyl tridecanoate	0.030 ± 0.002	0.030 ±0.002	0.030 ± 0.003	0.03 0 ± 0.002	0.010 ± 0.001	0.040 ± 0.003
Ethyl x-tetradecenoate	0.040 ± 0.02	0.10 ± 0.02	0.05 ± 0.01	0.22 ± 0.02	0.06 ± 0.01	0.12 ± 0.03
Ethyl y-tetradecenoate	0.030 ± 0.002	0.050 ± 0.003	0.030 ± 0.003	0.040 ± 0.002	0.01 ± 0.01	0.020 ± 0.003
Ethyl tetradecanoate	0.49 ± 0.03	0.75 ± 0.02	0.46 ± 0.03	0.65 ± 0.04	0.42 ± 0.03	0.60 ± 0.03
Ethyl pentadecenoate	0.050 ± 0.003	0.090 ± 0.003	0.06 ± 0.03	0.070 ± 0.003	0.030 ± 0.002	0.050 ± 0.002
Ethyl pentadecanoate	0.030 ± 0.003	0.070 ± 0.002	0.030 ± 0.002	0.030 ± 0.001	0.010 ± 0.002	0.040 ± 0.002
Ethyl 9-hexadecenoate	0.350 ± 0.002	0.42 ± 0.03	0.33 ± 0.03	0.52 ± 0.27	0.31 ± 0.02	0.44 ± 0.02
Ethyl x-hexadecenoate	n.d.	0.040 ± 0.002	0.010 ± 0.001	n.d.	n.d.	0.010 ± 0.002
Ethyl hexadecanoate	1.39 ± 0.01	1.86 ± 0.04	1.03 ± 0.05	1.19 ± 0.02	0.73 ± 0.03	0.87 ± 0.02
Ethyl 9,12-octadecadienoate	1.41 ± 0.03	0.32 ± 0.03	0.95 ± 0.05	1.23 ± 0.03	0.53 ± 0.03	1.76 ± 0.03
Ethyl linolenate	1.11 ± 0.04	2.09 ± 0.08	0.88 ± 0.02	1.02 ± 0.04	0.61 ± 0.03	0.58 ± 0.03
Ethyl (*Z*)-9-octadecenoate	0.03 ± 0.002	0.050 ± 0.003	0.020 ± 0.001	0.030 ± 0.003	0.020 ± 0.003	0.030 ± 0.002
Ethyl octadecanoate	0.050 ± 0.002	0.320 ± 0.02	0.020 ± 0.002	0.020 ± 0.002	0.010 ± 0.001	0.030 ± 0.003
2-ethylhexyl diphenyl phosphate	0.010 ± 0.001	n.d.	0.020 ± 0.001	n.d.	n.d.	0.020 ± 0.002
Total esters	23.12 ± 1.78 ^bc^	20.92 ± 1.18 ^d^	17.44 ± 0.69 ^e^	29.10 ± 1.28 ^a^	22.90 ± 1.08 ^c^	23.40 ± 1.54 ^b^
Average	20.49 ± 0.78 ^B^			25.18 ± 1.15 ^A^		

* data are presented as mean ± SD of three independent determinations; S1 (SGB vintage 2011), S2 (SGB vintage 2012), S3 (SGB vintage 2013), M1 (MGB vintage 2011), M2 (MGB vintage 2012), M3 (MGB vintage 2013); ** compounds in PCA analysis; n.d. = not detected; different superscript small letters in the same row indicate significantly different means (*p* < 0.05) for type of brandy from different vintages; different superscript capital letters indicate significantly different means (*p* < 0.05) for type of brandy.

**Table 3:** Concentration of terpenes in standard and Muscat grape brandies in mg/L *.

Aroma Compound (mg/L)	Standard Grape Brandy (SGB)			Muscat Grape Brandy (MGB)		
	S1	S2	S3	M1	M2	M3
*c*-linalool oxide	0.040 ± 0.003	0.010 ± 0.001	0.010 ± 0.001	0.30 ± 0.03	0.070 ± 0.003	0.020 ± 0.001
*t*-linalool oxide	0.010 ± 0.001	n.d.	n.d.	0.090 ± 0.002	0.030 ± 0.001	0.01 ± 0.02
Linalool **	0.06 ± 0.01 ^d^	0.050 ± 0.003 ^d^	0.050 ± 0.004 ^d^	0.81 ± 0.04 ^a^	0.41 ± 0.04 ^b^	0.28 ± 0.02 ^c^
alpha-terpineol **	0.05 ± 0.04	0.02 ± 0.02	0.010 ± 0.002	0.26 ± 0.37	0.12 ± 0.17	0.03 ± 0.02
2-propyl-1-heptanol	0.01 ± 0.01	0.10 ± 0.01	0.020 ± 0.002	0.010 ± 0.003	0.010 ± 0.001	0.030 ± 0.002
Citronellol **	0.08 ± 0.01 ^c^	0.030 ± 0.002 ^d^	0.040 ± 0.003 ^d^	0.24 ± 0.021 ^a^	0.11 ± 0.01 ^b^	0.080 ± 0.003 ^c^
Carvacrol, methyl ether	0.09 ± 0.01	0.030 ± 0.002	0.020 ± 0.002	0.20 ± 0.03	0.030 ± 0.003	0.02 ± 0.01
Geraniol **	0.010 ± 0.001 ^d^	0.010 ± 0.001 ^d^	0.010 ± 0.001 ^d^	0.31 ± 0.03 ^a^	0.13 ± 0.01 ^b^	0.08 ± 0.003 ^c^
Vitispirane	0.030 ± 0.003	0.020 ± 0.002	0.020 ± 0.002	0.13 ± 0.02	0.030 ± 0.003	0.020 ± 0.001
Carvacrol	0.010 ± 0.001	0.010 ± 0.002	0.010 ± 0.003	0.040 ± 0.003	0.010 ± 0.001	0.010 ± 0.001
(*E*)-beta-damascenone	0.020 ± 0.003	0.020 ± 0.002	0.020 ± 0.002	0.06 ± 0.01	0.020 ± 0.002	0.03 ± 0.01
*E*-nerolidol **	0.10 ± 0.02 ^d^	0.10 ± 0.02 ^d^	0.23 ± 0.02 ^c^	0.38 ± 0.03 ^a^	0.34 ± 0.03 ^ab^	0.31 ± 0.03 ^b^
(*2Z*,*6E*)-farnesol **	0.22 ± 0.03 ^a^	0.15 ±0.02 ^b^	0.12 ± 0.02	0.24 ± 0.02	0.25 ± 0.03	0.22 ± 0.03
13-epi-manool oxide	0.040 ± 0.002	0.040 ± 0.002	0.040 ± 0.002 ^b^	0.080 ± 0.003 ^a^	0.040 ± 0.002 ^a^	0.040 ± 0.003 ^a^
Total terpens	0.77 ± 0.09 ^c^	0.59 ± 0.01 ^c^	0.60 ± 0.02 ^c^	3.15 ± 0.52 ^a^	1.60 ± 0.14 ^b^	1.18 ± 0.05 ^bc^
Average	0.65 ± 0.08 ^B^			1.98 ± 0.16 ^A^		

* data are presented as mean ± SD of three independent determinations; S1 (SGB vintage 2011), S2 (SGB vintage 2012), S3 (SGB vintage 2013), M1 (MGB vintage 2011), M2 (MGB vintage 2012), M3 (MGB vintage 2013); ** compounds in PCA analysis; n.d. = not detected; different superscript small letters in the same row indicate significantly different means (*p* < 0.05) for type of brandy from different vintages; different superscript capital letters indicate significantly different means (*p* < 0.05) for type of brandy.

**Table 4:** Concentration of volatile acids in standard and Muscat grape brandies in mg/L *.

Aroma Compound (mg/L)	Standard Grape Brandy (SGB)			Muscat Grape Brandy (MGB)		
	S1	S2	S3	M1	M2	M3
Hexanoic acid **	0.59 ± 0.02 ^c^	0.53 ± 0.89 ^cd^	0.51 ± 0.87 ^d^	0.78 ± 0.85 ^a^	0.68 ± 1.06 ^b^	0.56 ± 0.94 ^e^
Octanoic acid **	0.86 ± 0.02 ^c^	0.80 ± 0.05 ^cd^	0.77 ± 0.03 ^d^	1.03 ± 0.04 ^a^	0.94 ± 0.03 ^b^	0.66 ± 0.05 ^e^
Decanoic acid **	2.82 ± 0.05 ^a^	1.58 ± 0.05 ^d^	1.93 ± 0.03 ^c^	1.98 ± 0.02 ^c^	2.42 ± 0.02 ^b^	1.33 ± 0.02 ^e^
Dodecanoic acid **	0.67 ± 0.03 ^bc^	0.49 ± 0.02 ^d^	0.47 ± 0.03 ^d^	0.97 ± 0.05 ^a^	0.72 ± 0.04 ^b^	0.62 ± 0.03 ^c^
Tetradecanoic acid	0.15 ± 0.02	0.20 ± 0.02	0.18 ± 0.02	0.31 ± 0.02	0.17 ± 0.02	0.14 ± 0.02
Hexadecenoic acid	0.09 ± 0.02	0.050 ± 0.002	0.060 ± 0.003	0.05 ± 0.03	0.060 ± 0.003	0.210 ± 0.002
Hexadecanoic acid **	0.50 ± 0.03 ^a^	0.30 ± 0.04 ^c^	0.50 ± 0.04 ^a^	0.45 ± 0.05 ^ab^	0.400 ± 0.002 ^b^	0.460 ± 0.002 ^ab^
9,12-octadecadienoic acid (*Z*,*Z*)	0.170 ± 0.002	0.21 ± 0.04	0.18 ± 0.02	0.12 ± 0.02	0.090 ± 0.003	0.20 ± 0.002
Octadecenoic acid, (*E*)-	0.21 ± 0.03	0.18 ± 0.02	0.16 ± 0.02	0.13 ± 0.02	0.13 ± 0.02	0.240 ± 0.002
Total vol. acid	6.06 ± 0.93 ^b^	4.34 ± 0.91 ^ab^	4.76 ± 1.13 ^ab^	5.82 ± 0.81 ^ab^	5.61 ± 1.12 ^ab^	4.42 ± 0.97 ^b^
Average	5.05 ± 1.00			5.28 ± 1.07		

* data are presented as mean ± SD of three independent determinations; S1 (SGB vintage 2011), S2 (SGB vintage 2012), S3 (SGB vintage 2013), M1 (MGB vintage 2011), M2 (MGB vintage 2012), M3 (MGB vintage 2013); ** compounds in PCA analysis; n.d. = not detected; different superscript small letters in the same row indicate significantly different means (*p* < 0.05) for type of brandy from different vintages.

**Table 5:** Concentration of aldehydes, acetals, and other compounds in standard and Muscat grape brandies in mg/L *.

Aroma Compound (mg/L)	Standard Grape Brandy (SGB)			Muscat Grape Brandy (MGB)		
	S1	S2	S3	M1	M2	M3
Aldehydes						
Acetaldehyd	210 ± 9	197 ± 8	221 ± 8	218 ± 9	199 ± 9	229 ± 9
Benzaldehyde **	0.19 ± 0.02 ^b^	0.25 ± 0.04 ^a^	0.20 ± 0.03 ^b^	0.18 ± 0.03 ^b^	0.17 ± 0.03 ^b^	0.17 ± 0.02 ^b^
Nonanal **	0.030 ± 0.004 ^c^	0.020 ± 0.002 ^d^	0.020 ± 0.003 ^d^	0.09 ±0.01 ^a^	0.050 ± 0.003 ^b^	0.05 ± 0.01 ^b^
Furfural	0.140 ± 0.003	0.34 ± 0.03	0.50 ± 0.03	0.29 ± 0.05	0.49 ± 0.02	0.13 ± 0.02
Total aldehydes	210.36 ± 77.95 ^bc^	197.61 ± 7.99 ^c^	221.72 ± 3.99 ^ab^	218.56 ± 8.16 ^ab^	199.71 ± 8.69 ^c^	229.35 ± 10.39 ^a^
Averages	209.90 ± 12.04			215.87 ± 15.20		
Acetals						
1,1-diethoxy-2-methylpropane	0.12 ± 0.03	0.150 ± 0.01	0.11 ± 0.02	0.10 ± 0.02	0.08 ± 0.02	0.10 ± 0.02
3,3-diethoxy-2-butanone	0.11 ± 0.02	0.070 ± 0.002	0.060 ± 0.004	0.18 ± 0.01	0.04 ± 0.01	0.010 ± 0.001
1-(1-ethoxyethoxy)pentane	0.21 ± 0.02	0.130 ± 0.002	0.13 ± 0.02	0.15 ± 0.03	0.16 ± 0.02	0.21 ± 0.02
Total acetals	0.44 ± 0.05 ^a^	0.35 ± 0.01 ^b^	0.30 ± 0.05 ^bc^	0.42 ± 0.02 ^a^	0.28 ± 0.02 ^c^	0.32 ± 0.04 ^bc^
Averages		0.36 ± 0.07			0.34 ± 0.07	
Other compounds						
Cyclohexane, ethoxy-	0.040 ± 0.003	0.040 ± 0.002	0.040 ± 0.002	0.11 ± 0.02	0.04 ± 0.02	0.06 ± 0.01
2-pentadecanone, 6,10,14-trimethyl-	0.10 ± 0.09	0.090 ± 0.002	0.090 ± 0.003	0.080 ± 0.002	0.05 ± 0.01	0.050 ± 0.003
Hexacosane	0.010 ± 0.001	0.010 ± 0.002	0.010 ± 0.003	0.050 ± 0.003	0.010 ± 0.001	0.020 ± 0.001
Heptacosane	0.010 ± 0.001	0.010 ± 0.002	0.010 ± 0.002	0.020 ± 0.002	0.010 ± 0.001	0.010 ± 0.003
Total	0.16 ± 0.02 ^b^	0.15 ± 0.001 ^bc^	0.15 ± 0.001 ^bc^	0.26 ± 0.02 ^a^	0.11 ± 0.01 ^d^	0.14 ± 0.01 ^c^
Average	0.15 ± 0.01 ^B^			0.17 ± 0.01 ^A^		

* data are presented as mean ± SD of three independent determinations; S1 (SGB vintage 2011), S2 (SGB vintage 2012), S3 (SGB vintage 2013), M1 (MGB vintage 2011), M2 (MGB vintage 2012), M3 (MGB vintage 2013); ** compounds in PCA analysis; n.d. = not detected; different superscript small letters in the same row indicate significantly different means (*p* < 0.05) for type of brandy from different vintages; different superscript capital letters indicate significantly different means (*p* < 0.05) for type of brandy.

**Table 6:** Sensory analyses of grape brandies (average values ± STD).

Sample	Colour (max 1)	Clarity (max 1)	Distinction (max 2)	Odour (max 6)	Taste (max 10)	Total (max 20)
Standard Grape Brandy (SGB)						
2011	1.0	1.0	1.82 ± 0.02	5.68 ± 0.17	9.48 ± 0.20	19.0 ± 0.16
2012	1.0	1.0	1.87 ± 0.03	5.70 ± 0.16	9.51 ± 0.18	19.1 ± 0.10
2013	1.0	1.0	1.80 ± 0.02	5.59 ± 0.20	9.46 ± 0.15	18.9 ± 0.15
Averages	1.0	1.0	1.83 ± 0.04	5.66 ± 0.06 ^b^	9.48 ± 0.02 ^a^	19.0 ± 0.10
Muscat Grape Brandy (MGB)						
2011	1.0	1.0	1.81 ± 0.02	5.75 ± 0.18	9.28 ± 0.20	18.8 ± 0.18
2012	1.0	1.0	1.83 ± 0.02	5.79 ± 0.16	9.37 ± 0.25	19.0 ± 0.00
2013	1.0	1.0	1.79 ± 0.01	5.69 ± 0.20	9.23 ± 0.20	18.7 ± 0.25
Averages	1.0	1.0	1.81 ± 0.02	5.74 ± 0.05 ^a^	9.29 ± 0.07 ^b^	18.8 ± 0.14

Different superscript small letters in the same row indicate significantly different means (*p* < 0.05) for type of brandy from different vintages.

## Data Availability

Not applicable.

## Sample Availability

Samples of the compounds are not available from the authors.

## References

[1] Lučić R. (1986). Production of Strong Alcoholic Beverages.

[2] Paunović R., Đurišić B. (1981). A contribution to the study of the method of production and properties of grape brandy. Vitic. Winemaking.

[3] Matijašević S., Bešlić Z., Przić Z., Žunić D., Todić S., Marković N., Ranković-Vasić Z., Ćirković B., Vukosavljević V., Ćirković D. (2016). Influence of cultivar characteristics of muscat table grapevine cultivars (Vitis vinifera L.) on grape brandy composition and quality. Ann. Univ. Craiova-Agric. Montanology Cadastre Ser..

[4] Paunović R., Nikićević N. (1988). Impact of grape variety on the composition and properties of grape brandy. Contemp. Agric..

[5] Nikićević N., Jović S., Sivčev B. Examination of the suitability of grape-based alcoholic beverages from some newly created grape varieties. Proceedings of the V Conference of the Industry of Alcoholic and Non-Alcoholic Beverages and Vinegar.

[6] Zhao Y.P., Li J.M., Xu Y., Fan W.L., Jiang W.G. (2009). Characterization of flavour compounds of four brandies by flavour extract dilution analysis. Am. J. Enol. Vitic..

[7] Vukoslavljević V., Ranković V., Žunić D., Matijašević S. (2015). Alcoholic fermentation influence on quality of grape brandies. Acta Agric. Serbica.

[8] The Official Gazette of Montenegro (2016). Law on Spirit Drinks of Montenegro br. 53/2016.

[9] Wei X.-F., Ma X.-L., Cao J.-H., Sun X.-Y., Fang Y.-L. (2018). Aroma characteristics and volatile compounds of distilled Crystal grape spirits of different alcohol concentrations: Wine sprits in the Shangri-La region of China. Food Sci. Technol..

[10] Crespo J., Rigou P., Romero V., García M., Arroyo T., Mariano Cabellos J. (2017). Effect of seasonal climate fluctuations on the evolution of glycoconjugates along the ripening period of grapevine cv. Muscat a petits grains blancs berries. J. Sci. Food Agric..

[11] Christoph N., Bauer-Christoph C. (2007). Flavour of Spirit Drinks: Raw Materials, Fermentation, Distillation and Ageing. Flavours and Fragrances.

[12] Tsakiris A., Kallithraka S., Kourkoutas Y. (2014). Grape brandy production, composition and sensory evaluation. J. Sci. Food Agric..

[13] Soufleros E.H., Mygdalia Ageliki S., Natskoulis P. (2004). Characterization and safety evaluation of the traditional Greek fruit distillate ‘‘Mouro’’ by flavor compounds and mineralanalysis. Food Chem..

[14] Apostolopoulou A.A., Flouros A.I., Demertzis P.G., Akrida-Demertzi K. (2005). Differences in concentration of principal volatile constituents in traditional Greek distillates. Food Control..

[15] Cortés S., Rodríguez R., Salgado J.M., Domínguez J.M. (2011). Comparative study between Italian and Spanish grape marc spirits in terms of major volatile compounds. Food Control..

[16] Spaho N. (2017). Distillation Techniques in the Fruit Spirits Production. Distillation—Innovative Applications and Modeling.

[17] Matijašević S., Popović-Djordjević J., Ristić R., Ćirković D., Ćirković B., Popović T. (2019). Volatile Aroma Compounds of Brandy ‘Lozovača’ Produced from Muscat Table Grapevine Cultivars (Vitis vinifera L.). Molecules.

[18] Hernández-Gómez F.L., Úbeda-Iranzo J., García-Romero E., Briones-Pérez A. (2005). Comparative production of diferent melon distillates. Chemical and sensory analyses. Food Chem..

[19] Lukić I., Milićević B., Banović M., Tomas S., Radeka S., Peršurić C. (2011). Secondary Aroma Compounds in Fresh Grape Marc Distillates as a Result of Variety and Corresponding Production Technology. Food Technol. Biotechnol..

[20] Plutowska B., Biernacka P., Wardencki W. (2010). Identification of Volatile Compounds in Raw Spirits of Different Organoleptic Quality. J. Inst. Brew..

[21] Silva M.L., Macedo A.C., Malcata F.X. (2000). Review: Steam distilled spirits from fermented grape pomace. Food. Sci Technol. Int..

[22] Cortés S., Gil M.L., Fernández E. (2005). Volatile composition of traditional and industrial Orujo spirits. Food Control..

[23] Raičević D., Pajović-Šćepanović R., Mijović S., Popović T. (2015). Phenolic compounds of red wines in Podgorica sub region (Montenegro). Agric. For..

[24] Pajović R., Raičević D., Popović T., Sivilotti P., Lisjak K., Vanzo A. (2014). Polyphenolic characterisation of Vranac, Kratošija and Cabernet Sauvignon (Vitis vinifera L. cv.) grapes and wines from dif-ferent vineyard locations in Montenegro. S. Afr. J. Enol. Vitic..

[25] Pajović-Šćepanović R., Raičević D., Wendelin S., Eder R. (2019). Characterization of the phenolic profile of commercial Montenegrin red and white wines. Eur. Food Res. Technol..

[26] Pajović-Šćepanović R., Krstić M., Savković S., Raičević D., Popović T. (2016). Wine quality in Montenegro. Agric. For..

[27] Raičević D., Božinović Z., Petkov M., Ivanova-Petropulos V., Kodžulović V., Mugoša M., Šućur S., Maraš V. (2017). Polyphenolic content and sensory profile of Montenegrin Vranac wines produced with different oenological products and maceration. Maced. J. Chem. Chem. Eng..

[28] Raičević D., Mijović S., Popović T., Pajović-Šćepanović R. The influence of variety and vintage on the chemical composition and sensory properties of red wines in Podgorica subregion (Montenegro). Proceedings of the 3rd International Symposium for Agriculture and Food—ISAF.

[29] Da Porto C., Sensidoni A., Battistutta F. (1995). Composition and flavour of muscat of canelli grape distillates obtained using different oenological techniques and unconventional distillation processes. Ital. J. Food Sci..

[30] Milanov G., Beleski K., Cvetković J., Nedelkovski D. (2014). Impact of grape variety and technological procedures on the quality of grape brandies. Agroznanje.

[31] The Official Gazette of Montenegro (2017). Rulebook on conditions regarding technical equipment and professional staff, sampling methods and physico-chemical analysis of. Off. Gaz. Monten..

[32] Publications Office of the European Union (2000). Commission Regulation (EC) No 2870/2000 of 19 December 2000 laying down Community reference methods for the analysis of spirits drinks. Off. J. Eur. Union.

[33] Ranković V., Palić R., Živković J., Mosić I., Stanković S., Stojanović G. (2004). Investigation of the impact of grape cultivars on the grape brandies quality. Phys. Chem. Technol..

[34] Tešević V., Nikićević N., Jovanović A., Djoković D., Vujisić L., Vučković I., Bonić M. (2005). Volatile Components of Plum Brandies. Food Technol. Biotechnol..

[35] Matijašević S., Todić S., Beslić Z., Ranković- Vasić Z. (2013). Volatile components of grape brandies produced from Muscat table grapevine (Vitis vinifera L.) cultivars. Bulg. J. Agric. Sci..

[36] Vulić T., Nikićević N., Stanković L., Veličković M., Todosijević M., Popović B., Urošević I., Stanković M., Beraha I., Tešević V. (2012). Chemical and Sensorial Characteristics of Fruit Spirits Produced from Different Black Currant (Ribes Nigrum L.) and Red Currant (Ribes Rubrum L.) Cultivars. Maced. J. Chem. Chem. Eng..

[37] Ledauphin J., Milbeau C.L., Barillier D., Hennequin D. (2010). Differences in the volatile compositions of French labeled brandies (Armagnac, Calvados, Cognac, and Mirabelle) using GC-MS and PLS-DA. J. Agric. Food Chem..

[38] Lukić I., Miličević B., Tomas S., Radeka S., Peršurić Đ. (2012). Relationship between volatile aroma compounds and sensory quality of fresh grape marc distillates. J. Inst. Brew..

[39] Arrieta-Garay Y., Blanco P., López-Vázquez C., Rodríguez-Bencomo J.J., Pérez-Correa J.R., López F., Orriols I. (2014). Effects of Distillation System and Yeast Strain on the Aroma Profile of Albarinño (Vitis vinifera L.) Grape Pomace Spirits. J. Agric. Food Chem..

[40] Geroyiannaki M., Komaitis M.E., Stavrakas D.E., Polysiou M., Athanasopoulos P.E., Spanos M. (2007). Evaluation of acetaldehyde and methanol in greek traditional alcoholic beverages from varietal fermented grape pomaces (Vitis vinifera L.). Food Control..

[41] Cacho J., Moncayo L., Palma J.C., Ferreiraa V., Culleréa L. (2013). Comparison of the aromatic profile of three aromatic varieties of Peruvian pisco (Albilla, Muscat and Torontel) by chemical analysis and gas chromatography-olfactometry. Flavour Fragr. J..

[42] Matias-Guiua P., Rodríguez-Bencomoa J.J., Pérez-Correab J.R., Lópeza F. (2018). Aroma profile design of wine spirits: Multi-objective optimization using response surface methodology. Food Chem..

[43] Schreier P., Drawert F., Winkler F. (1979). Composition of Neutral Volatile Constituents in Grape Brandies. J. Agric. Food Chem..

[44] Doneva-Šapčeska D., Dimitrovski A., Bojadžiev T., Milanov G., Vojnovski B. (2006). Free and potentially volatile monoterpenes in grape varieties from the Republic of Macedonia. Bull. Chem. Technol. Maced..

[45] Diéguez S.C., De La Peña M.L.G., Gómez E.F. (2003). Approaches to spirit aroma: Contribution of some aromatic compounds to the primary aroma in samples of orujo spirits. J. Agric. Food Chem..

[46] Herraiz M., Reglero J.G., Herraiz T., Loyolae E. (1990). Analysis of Wine Distillates Made from Muscat Grapes (Pisco) by Multidimensional Gas Chromatography and Mass Spectrometry. J. Agric. Food Chem..

[47] Vujović D., Maletić R., Popović-Đorđević J., Pejin B., Ristić R. (2016). Viticultural and chemical characteristics of Muscat Hamburg preselected clones grown. J. Sci. Food Agric..

[48] Bortoletto A.M., Carolina C.A., André R.A. (2016). Fatty acid profile and glycerol concentration in cachaças aged in different wood barrels. J. Inst. Brew..

[49] Publications Office of the European Union (2019). Regulation (EU) 2019/787 of the European Parliament and of the Council of 17 April 2019 on the definition, description, presentation and labelling of spirit drinks, the use of the names of spirit drinks in the presentation and labelling of other foodstuffs, the protection of geographical indications for spirit drinks, the use of ethyl alcohol and distillates of agricultural origin in alcoholic beverages, and repealing Regulation (EC) No 110/2008. Off. J. Eur. Union.

